# DLCDroid an android apps analysis framework to analyse the dynamically loaded code

**DOI:** 10.1038/s41598-025-88003-6

**Published:** 2025-01-26

**Authors:** Rati Bhan, Rajendra Pamula, K Susheel Kumar, Nand Kumar Jyotish, Prasun Chandra Tripathi, Parvez Faruki, Jyoti Gajrani

**Affiliations:** 1 School of Computing Science and Engineering, Galgotias University, Greater Noida, 203201 India; 2 Department of Computer Science and Engineering, Indian Institute of Technology (ISM), Dhanbad, 826004 India; 3 Department of Information Technology, Manipal Institute of Technology Bengaluru, Manipal Academy of Higher Education, Manipal, 560054 India; 4 Department of Computer Science & Engineering, Birla Institute of Technology, Mesra, Ranchi, Jharkhand India; 5 Department of Electrical and Computer Science Engineering, Institute of Infrastructure Technology Research and Management, Ahmedabad, Gujarat India; 6 Department Computer Science, University of Sheffield, Sheffield, United Kingdom; 7 Department of Technical Education, Govt. of Gujarat, Gandhinagar, India; 8 Department of Computer Science and Engineering, Engineering College Ajmer, Ajmer, Rajasthan India

**Keywords:** Dynamic Code, Reflection API, Android Malware, Application Security, Computer science, Information technology, Software

## Abstract

To combat dynamically loaded code in anti-emulated environments, DLCDroid is an Android app analysis framework. DL-CDroid uses the reflection API to effectively identify information leaks due to dynamically loaded code within malicious apps, incorporating static and dynamic analysis techniques. The Dynamically Loaded Code (DLC) technique employs Java features to allow Android apps to dynamically expand their functionality at runtime. Unfortunately, malicious app developers often exploit DLC techniques to transform seemingly benign apps into malware once installed on real devices. Even the most sophisticated static analysis tools struggle to detect data breaches caused by DLC. Our analysis demonstrates that conventional tools areill-equipped to handle DLC. DLCDroid leverages dynamic code interposition techniques for API hooking to expose concealed malicious behavior without requiring modifications to the Android framework. DLCDroid can unveil suspicious behavior that remains hidden when relying solely on static analysis. We evaluate DLCDroid’s performance using a dataset comprising real-world benign and malware apps from reputed repositories like VirusShare and the Google Play Store. Compared to state-of-the-art approaches, the results indicate a significant improvement in detecting sensitive information leaks, more than 95.6% caused by reflection API. Furthermore, we enhance DLCDroid’s functionality by integrating it with an event-based trigger solution, making the framework more scalable and fully automated in its analysis process.

## Introduction

**Fig. 1:**
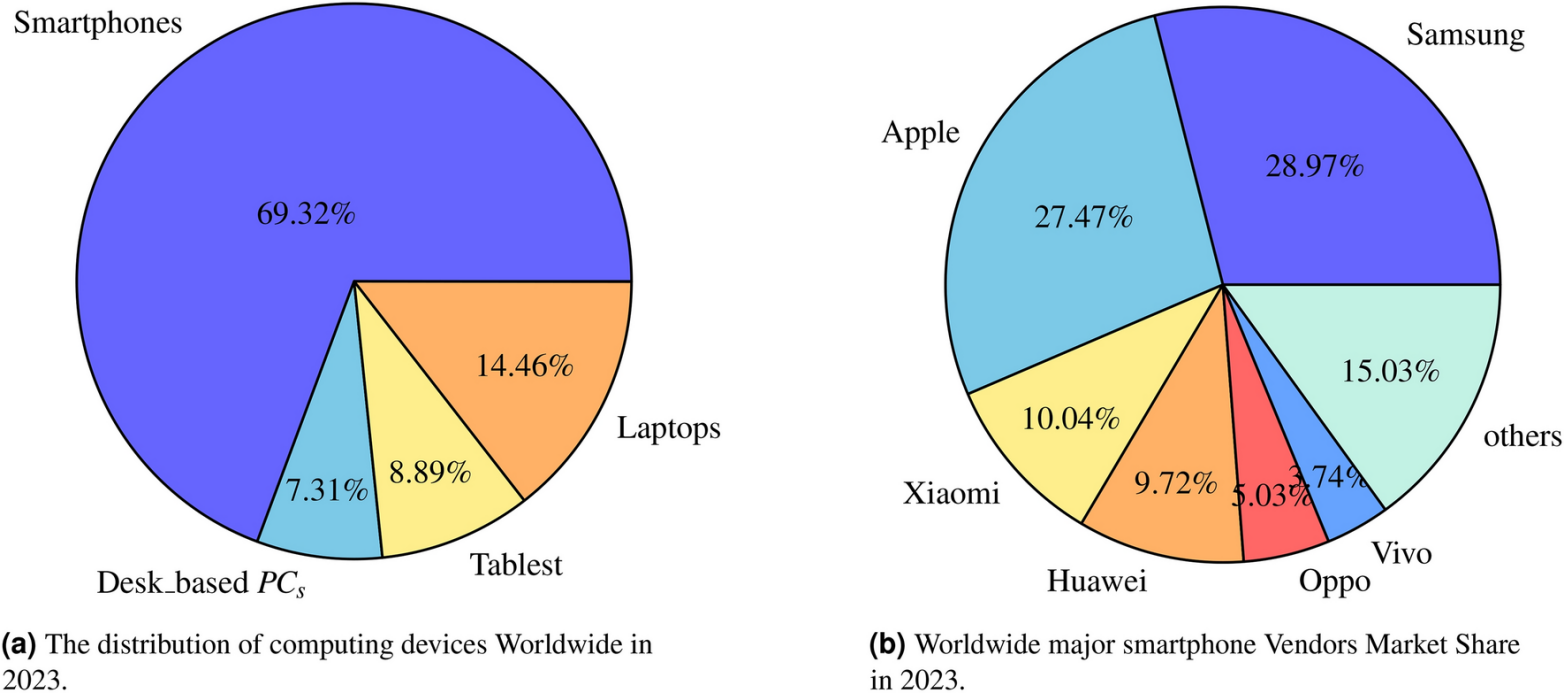
Smartphone Platform Status Worldwide in 2023.

**Fig. 2:**
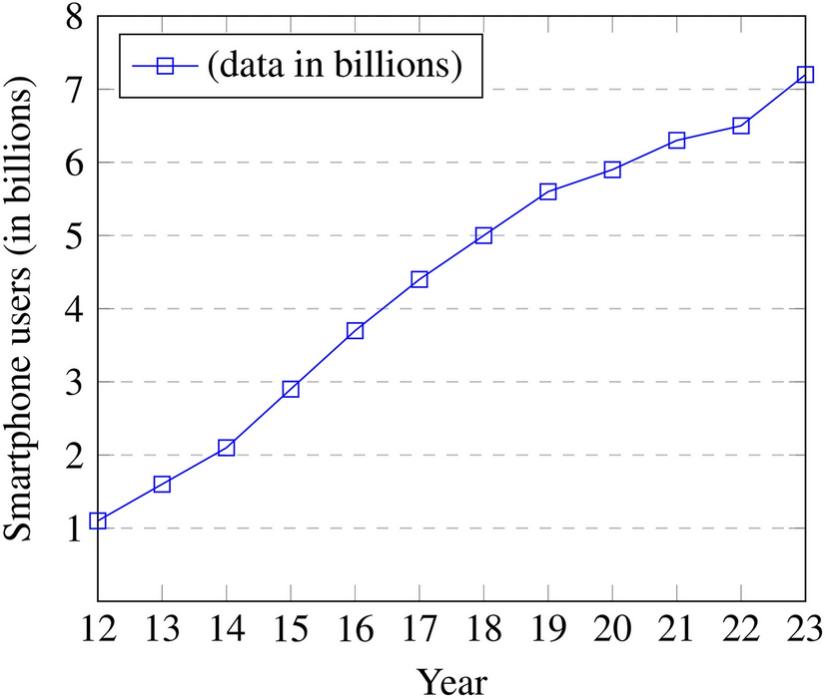
The number of smartphone users increased from 2012 to 2023.

The exponential increase among smart devices, especially Android smartphones, has become an essential part of everyday lives over the last decade. The inaugural Android phone, the “HTC Dream”,was formally launched on September 23, 2008. It was the inaugural handset to operate on Android, a smartphone OS released by Google. People have embraced intelligent devices coupled with high-speed Internet, enabling confidential personal and office data at the user’s fingertips. The smartphone is a unique digital platform enriching the ease and performance enhancement, thus improving overall user interaction. The Statista 2023^1^ reports indicate the growth of smartphone users from 1.06 billion to 7.2 billion between the years 2012 to 2023, as illustrated in Fig. 2. A recent report shows 1.75 billion smartphones sold in 2023, a more than ten-fold increase from 127 million units in 2007^2^. Around 4.5 million smartphone devices have been regularly activated every day, a tremendous amelioration of smartphone users. Fig. 1a indicates the uses of smartphones ($69\%$of overall electronic communicating) against laptops, desk-based PC systems^3^. It confirms that the study of MID security is essential^4^. Fig. 1b illustrates the importance of smartphone OS, especially the Android sky-rocketing to $70.7\%$ against the iOS global share reaches at $28.57\%$. However, the share of other smart-device OS is less than $1\%$in the year 2023. It emphasizing the importance of Android phone in the smartphone market^5^.

Applications can extend their functionality at runtime using dynamic code loading techniques like reflection and dynamic class loading. These capabilities were inherited from Java and have been incorporated into the Dalvik Virtual Machine (DVM), which is also supported by Android Runtime (ART), the successor to Dalvik. ART is responsible for executing applications and system services in Android, and it employs ahead-of-time (AOT) compilation to generate .oat files from DEX files. ART can run applications developed for the DVM and is backward compatible with the Dalvik platform. Dynamic Loaded Code (DLC) allows developers to load classes into memory during runtime. Similar to DVM, ART enables developers to load additional source code from remote servers, allowing apps to import files originating from external sources like the internet^6^. These files are stored in internal memory and contain valid classes.dex files within .apk, .jar, and .zip files. Android provides a hierarchy of class loaders to facilitate the transfer of classes into application memory.

Just as shared libraries assist programmers in building modular software, DLC empowers developers to enhance an app’s functionality by dynamically loading it from various sources, such as internal storage and the internet, at runtime. Some Android apps utilize dynamic updates retrieved from the internet, employing class loaders for this purpose. Rather than delivering updated versions, these apps offer incremental improvements in functionality. App updates are loaded dynamically from the internet using class loaders. While the current app may receive enhancements in functionality^7^, it doesn’t necessarily provide updated versions of the app. Apps can rely on common frameworks tailored to their specific functionality, like an ad-supported framework that displays ads to users. These common frameworks are installed as standalone applications, and dependent apps import their code dynamically. Without DLC, each dependent app would need to implement the framework’s functions individually. DLC allows the framework to be updated to enhance the underlying functionality for all dependent apps, rather than requiring updates to each individual app. The aim of this study aimed to address the subsequent research enquiries:

1. Research Question 1 (RQ1): Why is the dynamically loaded code technique more dangerous than other malware developers employ?
2. Research Question 2 (RQ2): To what extent do malware applications utilise downloadable content (DLC) to enhance their destructive capabilities?
3. Research Question 3 (RQ3): How can we initially categorise the malware apps with DLC functionality amidst the vast amount of malware developed daily?
4. Research Question 4 (RQ4): What is the additional execution cost necessary to assess malware applications that utilise DLC code to enhance their dangerous functionality?

Static analysis methods are ineffective to detect the malware that dynamically modifies application code to perform malicious actions. Notable static analysis frameworks such as AmanDroid^8^, IccTA^9^, DroidSafe^10^, DroidRA^11^, and FlowDroid^12^ are failed to identify data leaks resulting from dynamically loaded application code. Consequently, a significant necessity is to incorporate a reflection-aware dynamic analysis technique with ICC.

To tackle this difficulty, we proposed a framework named DLCDroid, which integrates static and dynamic evaluations of applications utilising DCL and reflection APIs. DLCDroid employs dynamic code interposition techniques for API hooking, allowing it to reveal undetectable malicious behaviour without altering the Android architecture. We test DLCDroid’s accuracy using a dataset from reputed benchmarks like VirusShare and the Google Play Store. The primary contributions of this work is encapsulated as follows:

1. Our analyzed dataset comprises a substantial collection of benign applications and malware samples obtained from multiple reputable repositories. Our findings indicate that a significant number of Android malware applications utilize dynamically loaded code to perform malicious activities.
2. We developed and executed DLCDroid, a system that integrates static and dynamic analysis to uncover concealed malicious activities. DLCDroid analyzes dynamically loaded code and resolves the targets of Reflection API, enhancing the application’s control flow graph with information acquired during static analysis. Consequently, DLCDroid can be utilized alongside additional static analyzers to enhance the precision of their analyses.
3. We assessed DLCDroid using a collection of actual apps. We observe that DLCDroid effectively identified malicious activities that was undetected during static analysis.

The remainder of the paper is structured as follows. Section 2 offers an overview of dynamic class loading and the reflection API, and illustrates various methods by which malware might use reflection code to perform malicious functionality. Section 3 delineates the issue statement and provides a comprehensive overview of DLCDroid, whilst Section 4 addresses the implementation specifics. Section 5 presents the analysis of results, while Section 6 addresses the limits of the existing implementation. Section 6 delineates the linked work, while Section 7 finishes the research and anticipates future endeavors.

## Background

**Fig. 3:**
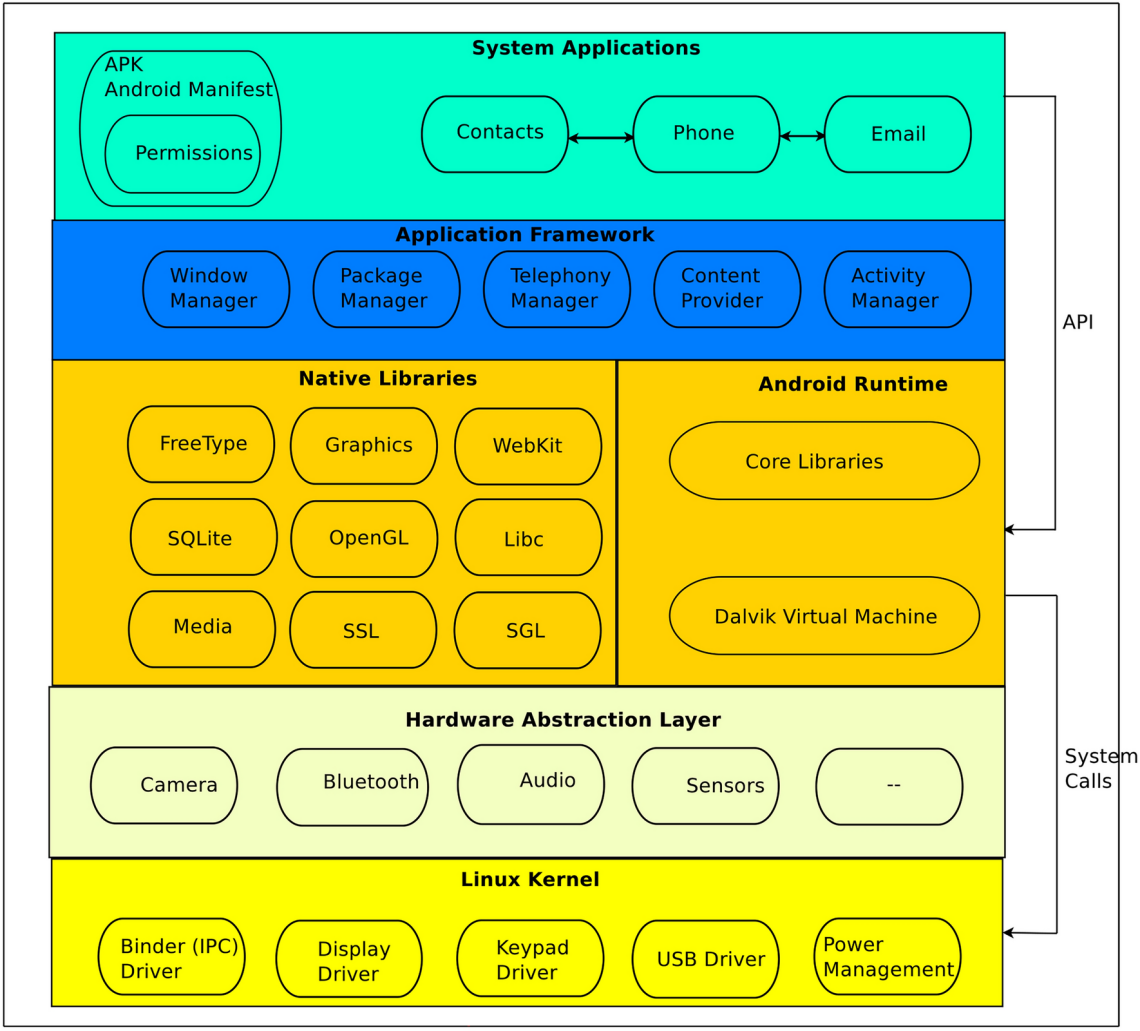
Android layered architecture.

Android is the most popular Operating System (OS) platform for mobile devices, a modified version of Linux OS as per resource constraints of mobile devices like lack of memory register, limited battery, and computation power over desktop machines. Some extra features added in Android like alarm driver, power management, kernel debugger, shared memory driver, and Android logger as Android Architecture is shown in Fig. 3. Android uses its C library known as Bionic for quick execution as compared to GNU C. Likewise; Android uses its Virtual Machine known as Dalvik Virtual Machine(DVM)^13^, which is later replaced by Android Run Time (ART). Furthermore, Android uses Yet Another Flash File System(YAFFS) in NAND-type flash memory to fast read/write access compared to a hard disk drive. Android app is mainly written in the java language known as Android apps. Apps developers use an extensive collection of Application Programming Interfaces (APIs) regulated by the Android Software Development Kit (SDK). The archive file combined Apps bytecode and resources named Android Application Package (APK) and executed in a secured environment known as Sandbox using ART environment^14^.

### Android application

Android apps have four components named Activity, Service, Content Provider, and Broadcast-receiver. The Activity component is used to provide a user interface to the user, and the Service component is used to provide process execution in the background, not interact with the user^15^, Content Provider uses to provide the data to apps which are shared across multiple apps. Finally, the Broadcast-receiver is mainly used to announce messages system-wide.

#### Application configuration

AndroidManifest.xml is the mandatory configuration file for every app. It specifies the principal component which constitutes the apps, along with their types, requirement, capabilities, and permission needed^16^. All the values defined in the manifest file are given at the compile time of apps. Therefore, the user cannot modify them during run-time.

#### Inter-Component communication

Android isolates and distributes system resources by a sandbox mechanism to provide protection. Android apps interact with other apps by message passing, known as Inter-component communication (ICC). Android apps use ICC mainly for Intent messages. Set of Intent-Filters used to specify component capabilities to respond to its kind of requests^17^. The Intent specifies the action performed by the event and the required data. There are many ways to invoke components like implicit or explicit, inter-apps or intra-apps. Android’s ICC provides late binding or run-time binding within the same or among multiple apps. Android apps can call the components by event messaging, an essential property of an event-driven system, while such calls are not mentioned explicitly in its code. There are many security issues introduced by researchers based on the mechanism of ICC^18^. For example, it is possible to temper or intercepts the message of the Intent event since authentication or encryption is not applied^19,20^. Android has no mechanism to prevent the intention of misrepresenting the third party ICC caller to its callee^21^.

#### Permissions

Apart from sandboxing, imposing permissions is also a good mechanism to protect Android apps. Android permissions mechanism is a pillar of Android app security. The permissions define in the manifest file allow access to sensitive resources and interaction with other apps in a secure way^22^^23^. The Android system asks for user authorization of requested permissions at app installation. Users can refuse the grant of requested permission and cancel the app installation. Along with required permissions, the app may impose those permissions held by other Android apps and have the permission to interact with such apps. Apart from built-in Android permission, any app can define its permissions to self-protection of different system resources. The current model of Android permission has a shortcoming, discussed by various researchers^24^. For example, some defects occurred due to permission violation of the least privilege principle^25^. Delegation attack due to access control permission at individual app^26^^25^. Some attacks are performed due to uninformed decisions of end-users having a lack of awareness of Android permissions^27^^28^.

### Android layered architecture

As depicted in Fig. 3, the Android smartphone OS is based on Linux with some modifications due to resources constrained environment. The functionality of Android OS is based on many layers explained as follows.

**Kernel:** It is the lowest layer responsible for managing system components like network, memory, process and device security, and so on.

**Hardware Abstraction Layer (HAL):** It serves as a communication link between the apps and device drivers of specific hardware components like Bluetooth, camera, GPS, and many more. The way HAL is implemented differs from one manufacturer to the next.

**Virtual Machine (VM):** Android uses two types of virtual machines (VMs) to execute apps in a protected environment, known as Sandboxing. In later Android versions 5.0 onwards, ART is recognized advanced execution environment, whereas all preceding versions of Android use DVM. VM delivers excellent run-time performance by optimizing garbage collection and reducing power consumption.

**Libraries:** Native C/C++ libraries are used to provide essential system services and Android components such as HAL, DVM, and ART. Several libraries (Inbuilt API) are available to help build an app’s user interface, graphics drawing, and database access.

**Apps development tools:** Android Studio and SDK provide a complete collection of development tools as well as a Java-based API for developing Android apps. The debugger, QEMU-based emulator, and sample code repository are helpful tools.

**Application layer:** This layer is found at the top of the stack and includes both system native apps that provide essential functions like web surfing, messaging, email composition, and audio/video calling, as well as third-party apps, downloaded and installed from various App Stores by the user.

### Implications of reflection API in android apps

Reflection API refers to a JAVA program’s ability to treat its code as data and modify it while running^29^. It enables the JAVA program to introspect and alter its behaviour during runtime dynamically. Reflection plays a crucial role in Android application development, offering the flexibility to avoid static inspection. Most researchers have made efforts to analyze Android apps using Reflection API. Android apps leverage Java’s reflection APIs extensively for obtaining Class objects, inspecting and creating instances of classes, modifying Class members, and invoking their methods. All these characteristics also attract malware developers’ attention to gain information about app code during execution.

In app analysis, a “sink” refers to any method that can expose user data. At the same time, a “source” denotes any mechanism that reveals the user’s sensitive information using Reflection API. Android apps utilize reflection APIs to obscure the interactions with critical sources and sinks. We explained the reflection APIs categorized into five main groups^30^, accompanied by examples illustrating how each category can address sensitive information leakage like User Communications Details, Contact Information, Financial Information, Identification Information, Authentication Credentials, Device Information IMEI number etc. while executing apps.

#### Creating an instance of the class and determining the class name of an object.

The reflection APIs can instantiate a class that matches the members of sensitive sources and sinks. In this section, We demonstrate how to get and leak Device IMEI number using Reflection API, which has never been statically declared in the source code and cannot be determined by static string inference.

For instance, in the MainActivity, an object is provided through the newInstance() API, and to obtain an object of BaseClass, the method forName() is used, followed by accessing the field Str using the getField() method. Taint analysis is used to recognize the instantiation of the class and distinguish between sinks and sources appropriately.

**Listing 1:**
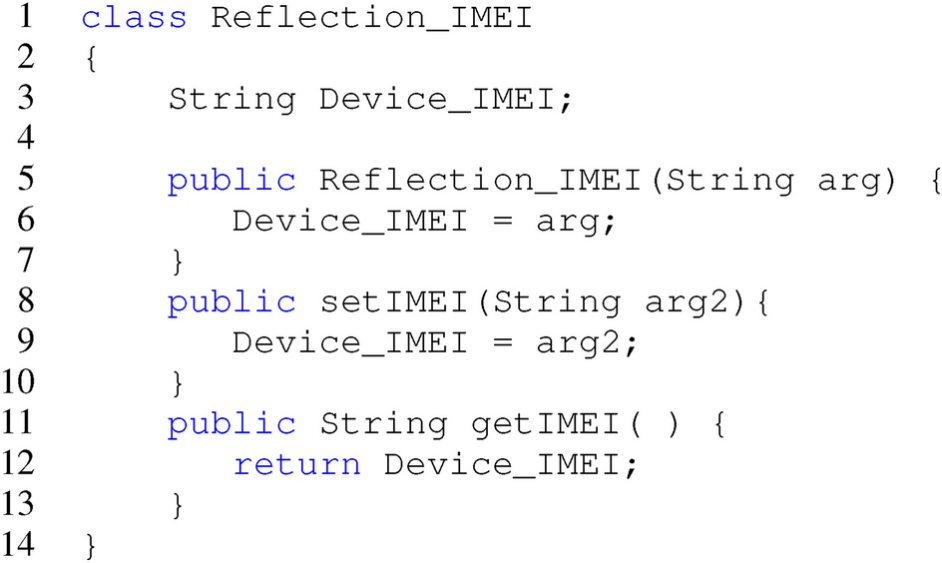
Accessing Device’s IMEI.

In the code snippet shown in Listing 1 Accessing Device’s IMEI, the class Reflection_IMEI has data member Device_IMEI, arg is a single argument of the constructor, and two methods setIMEI() and getIMEI().

**Listing 2:**
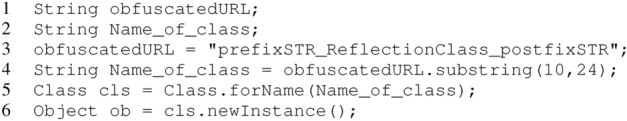
Create object of the Reflective class.

The code snippet shown in Listing 2 dynamically loaded the class ReflectionClass using Class.forName(Name-_of_class) method and create an object of loaded class ob using newInstance() method of reflection API. The code clarifies that the real class name, ReflectionClass, passed as an argument of the reflection API.

#### Call and misuse the methods of sensitive classes

This section discussed how to load the code dynamically from a remote server and call the method of sensitive classes (e.g., get and leak Device IMEI number ) using Reflection API and thwart taint analysis techniques. This technique illustrates an attempt to obfuscate the flow of sensitive information using reflection API, making it challenging for static analysis tools to detect and analyze such behaviour. The methods of java.lang.reflect class, such as invoke() and getMethod(), are employed to invoke a method and thwart taint analysis dynamically. In the getMethod() method of java.lang.reflect class, the first argument provided is the method identifier to be called dynamically, followed by an array specifying the method arguments. The getField() and newInstance() methods of the reflection API are used to obtain the field and create an object of the dynamically loaded class. Additionally, it utilizes the invoke() methods of the reflection API to retrieve and execute the methods of the dynamically loaded class.

**Listing 3:**
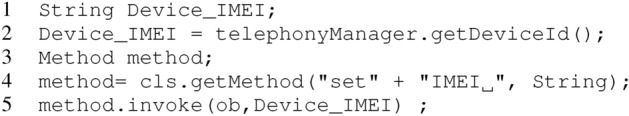
Dynamically call the method using Reflection API.

The device’s IMEI is retrieved and stored in the variable Device_IMEI in Listing 3 using the reflection API method getDeviceId(). When this IMEI is used as a source for any potential data sink, a taint analysis technique is activated to mark the Device_IMEI variable as tainted, thereby detecting the potential information leak from the device. To evade this taint analysis, the sample code attempts to subvert the analysis by storing this information in another field using reflection. This approach aims to prevent the proliferation of taint markings.

The ob variable holds an object of the ReflectionClass after being initialized through the reflection API. Then, utilizing the getMethod() method, the ReflectionClass object is used to create a method called setIMEI(string), where the subsequent parameter is specified as a String since the method expects arguments of type String. The invoke() method is subsequently invoked with two arguments, ob and Device_IMEI, to call the setIMEI(String) method dynamically. Significantly, the identifier and signature of the setIMEI(String) method cannot be determined statically from the source code alone.

#### Access and set the fields of dynamically loaded classes

This section discussed how to access and change the value of the fields of the dynamically loaded classes using Reflection API. The Java java.lang.reflect APIs utilized for setting and retrieving the contents of class fields. The Reflection APIs enable access to the specified data members of a dynamically loaded class in the form of field objects through methods getField() and getDeclaredField().

**Listing 4:**
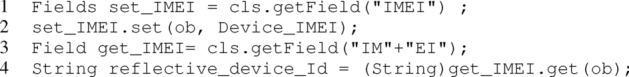
Access and set the fields of dynamically loaded classes to thwart taint analysis.

In the code snippet presented in Listing 4, field reflection APIs are employed to prevent the propagation of taint analysis. The code assigns the value of Device_IMEI to the field IMEI using the set_IMEI field object of the ReflectionClass.

The IMEI field is created as a field object called get_IMEI, which is utilized to retrieve the value and place it in the field named reflective_device_Id. Since the field name, IMEI, is not statically resolved, this small piece of code effectively halts the static taint from spreading while reflectively storing the value of Device_IMEI in reflective_device_Id. To conceal the device information leak, it forwards reflective_device_Id to the sink rather than Device_IMEI.

#### Getting and instantiating constructor of dynamically loaded classes

This section discussed how to get and instantiate the constructor of dynamically loaded classes using Reflection API. The constructor of the dynamically loaded class retrieved the Device_IMEI. Subsequently, the IMEI number of the device is sent as an SMS using SmsManager. The traditional assessment methods cannot detect such information leaks through reflection API.

Using the Reflection API forName(), we can dynamically load the ReflectionClass, create its object through the newInstance() API, retrieve its constructor with the getConstructor() API, and then access its variables by employing the getField() API. The getConstructor() API is used to obtain the constructor based on the specified argument types. The constructor’s object is returned if it matches the defined parameters of the argument. The object, obtained through the newInstance() API, can be used to invoke the constructor. Malicious apps exploit constructors within the class field to access confidential information and reveal device details.

**Listing 5:**
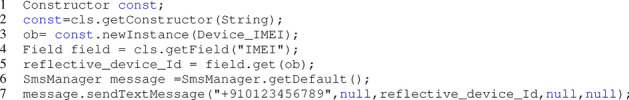
Instantiate Constructor to leak device information through Reflection API.

In the example presented in Listing 5, an instance of the constructor of ReflectionClass is created using the reflection API. The constructor is then invoked with Device_IMEI as an argument. The constructor within the ReflectionClass object ob stores the Device_IMEI. Subsequently, with the assistance of the field reflection API, the IMEI is retrieved and sent as an SMS using SmsManager. An effective assessment should be capable of handling the reflection API to detect such information leaks.

#### Leak sensitive information using intent reflection API

This section demonstrated how to leak sensitive information using the Intent Reflection API. The Reflection APIs can dynamically load the code and leak sensitive information in various ways, which is relatively straightforward to implement. More complex scenarios, like encryption, array subscripts, unresolved intents, concatenation, and substring operations, are employed to evade static analysis. Additionally, reflection enables the invocation of intents, classes, and methods and performs malicious activity. A different approach that demonstrates the invocation of intents using Reflection API is illustrated in Listing 6.

**Listing 6:**
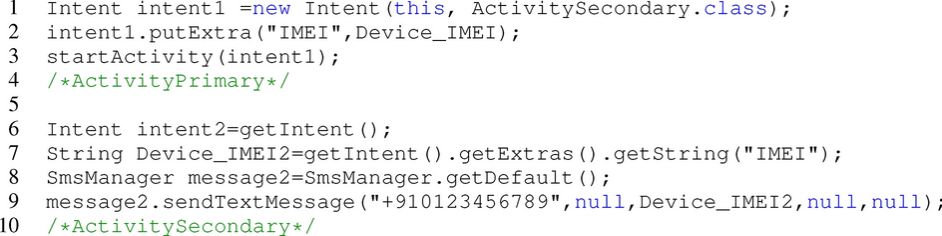
Leak phone IMEI number using Intent Reflection API.

The ICC and Intent Reflection APIs in Listing 6 demonstrate how to expose the phone’s IMEI number. ActivityPrimary employs Intent (Intent1) to send the Device_IMEI number, while ActivitySecondary uses Intent (Intent2) to receive it as a parameter named IMEI. The Device_IMEI2 information is leaked by ActivitySecondary through the use of the sendTextMessage API.

## Problem description and proposed solution: DLCDroid

Nowadays, smartphones have become an integral part of life and act as personal assistants, carrying the user’s sensitive information like Device Information, Personal Identification Information (PII), Contact Information, Location Data, Communications, Financial Information, Authentication Credentials, Multimedia, Health Information, Browsing History and App Usage etc. in various forms like short message service (SMS), audio and video recording, voice calls, Device and Network Details and image capturing. Android Smartphone’s popularity among users and developers is due to its open architecture and third-party apps. Third-party developers can update the app to introduce malicious activities. Smartphone security is an active area of research that effectively identifies information leaks within malicious apps, where malware developers continuously involve new innovative techniques like reflection and code obfuscation.

Applications can extend their functionality at runtime using Dynamic Loaded Code (DLC) techniques, such as dynamic class loading and reflection. Unfortunately, malicious app developers often exploit these techniques to transform seemingly benign apps into malware once installed on real devices. Even the most advanced static analysis tools struggle to detect data breaches resulting from DLC code. Our analysis demonstrates that conventional tools need to be equipped to handle apps containing DLC components. Malware apps leverage intent reflection to obscure communication between source and sink components.

Our analysis of 50 Android apps, sourced from repository AndroZoo^31^, revealed that reflection is widely used with non-constant arguments in both benign and malicious apps. Static analysis techniques, such as FlowDroid^12^and DroidSafe^10^, were employed to identify reflection-related behaviors, and dynamic validation was performed in a sandboxed environment to observe runtime execution. We found that over 70% of reflective calls used dynamically constructed arguments, such as concatenated strings or runtime variables, which static string-based analysis could not detect effectively. These findings motivated the design of DLCDroid to overcome the limitations of reflection-aware static analysis.

By misusing reflection APIs, attackers obscure the path between source and sink methods. These attackers are well-versed in evasive strategies, particularly those involving reflection with parameter obfuscation and ICC. Static analysis approaches aware of reflection APIs typically do not uncover dependent leaks during app execution. In such cases, dynamic analysis proves to be beneficial. Our goal with dynamic analysis is to enhance the capabilities of modern static analyzers in detecting leaks driven by reflection that might otherwise remain unnoticed. The objectives of reflection APIs, such as execution time dependency and identification obfuscation, will aid in identifying all leaks caused by reflection.

Static analysis techniques struggle to identify malware that dynamically updates app code to execute malicious activities. Prominent static analysis frameworks like AmanDroid^8^, IccTA^9^, DroidSafe^10^, and FlowDroid^12^ fail to identify the demonstrated leak because reflection hinders taint analysis. Additionally, DroidRA^11^, a reflection-aware static assessment tool, does not detect the data leakage caused by dynamically loaded app’s code. DroidRA failure results from the dynamic construction of the app’s method name.

Therefore, there is a strong need for a reflection-aware dynamic analysis technique integrated with ICC. To address this challenge, the authors have introduced a framework called DLCDroid, which combines static and dynamic assessments of apps employing DCL and reflection APIs. DLCDroid leverages dynamic code interposition techniques for API hooking, enabling it to expose concealed malware behaviour without modifying the Android framework. We evaluate DLCDroid’s performance using a dataset comprising real-world apps from benchmarks like VirusShare and the Google Play Store. The results indicate a significant improvement in detecting sensitive information leaks caused by reflection compared to static analysis tools. DLCDroid can unveil suspicious behaviour that remains hidden when relying solely on static analysis.

The DLCDroid framework comprises two phases in the analysis process. Static analysis of an app occurs on the system, while during dynamic analysis, the app runs on an actual Android mobile phone or an Android emulator. The DLCDroid framework allows for the easy integration of any static analyzer, facilitating the work of analysts. The system’s static analyzer initially generates the application’s Control Flow Graph (CFG). The dynamic analysis results obtained from the app’s emulator execution are combined to perform a comprehensive analysis. Through API hooking techniques, the dynamic analysis component of DLCDroid monitors dynamic behaviour by intercepting calls to dynamically loaded code.

DLCDroid effectively integrates dynamic analysis and code instrumentation, as demonstrated in Listings 4.3 to 4.6, to handle execution dependencies and prevent static inference of details regarding the return values and arguments of reflection APIs. The purpose of including dynamic analysis is to gather such information by observing reflection APIs associated with the categories described in Section 2.3. Figure 3 illustrates the process diagram of our proposed DLCDroid. The steps of the DLCDroid approach are outlined below.

**Fig. 4:**
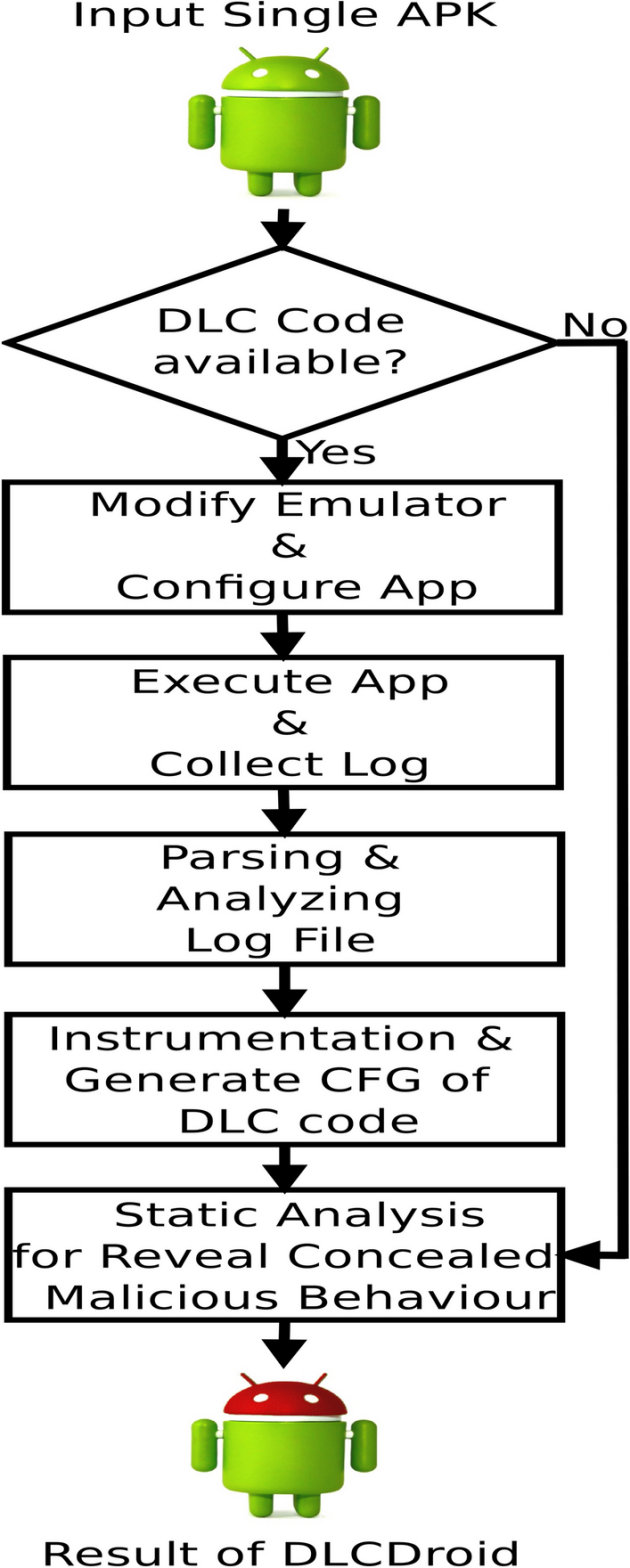
Methodology of Proposed DLCDroid Framework.

1. **Determine the Presence of DLC Code:** Verify whether DLC code exists within the application. If it does, proceed to DLCDroid analysis; otherwise, proceed with static analysis to identify information leaks. There are multiple ways to detect the presence of DLC code in apps are as follows:
  **Manual Code Review:** Perform an exhaustive manual inspection of the source code, methodically scrutinizing for any indicators of the utilization of Java’s Reflection API. Examine the code to identify the inclusion of classes and methods derived from the java.lang.reflect package, namely Class, Method, and Field. It is advisable to exercise vigilance in identifying certain keywords such as Class.forName, getField, getConstructor, and getMethod, as they may indicate the presence of Reflection API implementation.
  **Static Analysis Tools:** Utilise static code analysis tools to automate the identification of Reflection API utilisation inside the codebase. These tools are capable of inspecting the code for known patterns and method calls associated with reflection. Well-regarded tools for this purpose include AndroGuard, FindBugs, Project Management Professional (PMD), and Checkstyle. In addition, modern Integrated Development Environments (IDEs) may feature built-in code analysis capabilities designed to identify Reflection API usage.
  **Bytecode Analysis:** Utilize specialized bytecode analysis tools like Abstract Syntax Manipulation (ASM), Byte Code Engineering Library (BCEL), or Byte Buddy to scrutinize the compiled bytecode of your Java application. These tools demonstrate proficiency in identifying occurrences of reflective activities at the bytecode stage. It is important to acknowledge that this particular approach is more complex and requires a more comprehensive comprehension of the subtleties of bytecode.
  **Custom ClassLoader:** One possible approach to determining the utilization of the Reflection API involves the development of a customized class loader. The custom, as mentioned earlier, loader possesses the capability to intercept the process of loading classes and offer valuable insights pertaining to dynamically loaded classes. Although it may not directly identify the use of the Reflection API, it can shed light on dynamic class loading, which is frequently associated with reflective techniques.
  **Logging and Monitoring:** Implement a system for logging and monitoring within your application. By incorporating custom code to record instances of Reflection API usage during runtime, you can accumulate valuable data concerning where and how reflection is employed within your application. This approach serves to enhance your overall understanding of reflection utilization.
2. **Modify the Emulator and Configure the App:** Modify the emulator to evade the anti-emulation techniques employed by modern malware to identify emulated environments and evade detection. The app itself is configured using APIMonitor to capture all reflection APIs in the particular format specified within the Android logcat 4 during the execution of the application.
3. **Execute the App and Collect Logs:** Execute the Configured app on a modified emulator to intercept the dynamically loaded code during execution and capture every reflection API in the Android Logcat. The Logcat file contains the details regarding the methods being called and their arguments, including the stack-trace in the context of a reflection API.
4. **Parse and Analyze the Log File:** Parse the log file to determine the line number of each reflection API and searches for the two specified tags, WRAP_API and REF_CALL. Analyze the data collected from the log files and create app’s Control Flow Graph (CFG). Examines every downloaded file to determine if it contains additional Methods of Interaction (MOIs).
5. **Instrumentation and Transform the App Code:** DLCDroid generates instrumented apps using API hooking. Transform the app code by replacing the invocation of reflection methods with equivalent non-reflection APIs. We stack-trace information for both the reflection and DLC scenarios is used to determine which MOI initiated the call.
6. **Reveal Concealed Malicious Behaviour:** In the final phase, the transformed app undergoes static analysis to identify suspicious activities. The suspicious patterns, such as reflective calls to Android APIs protected by unsafe permissions and suspicious inputs, assist in identifying concealed malicious behavior via reflection APIs.

## Implementation

Fig.4 Depicts the step-by-step process of the DLCDroid framework. DLCDroid conducts a comprehensive analysis of the app’s code, focusing on instances of reflection and DCL techniques. It begins by creating a Control Flow Graph (CFG) of the app’s content. If no instances of reflection or DCL are found, DLCDroid terminates its analysis. However, if such instances are detected, DLCDroid engages a Python program to perform static analysis using the AndroGuard framework, which represents the app’s compiled code as a collection of manipulable Python objects. DLCDroid is compatible with most static analysis tools examining .dex and .apk files and enhances the identification of suspicious behavior by modifying the permission map in AndroGuard.

DLCDroid extracts the classes.dex file from the app and scans the code for instances of DLC and reflection calls. It then proceeds to modify an emulator to resist anti-emulation techniques and conducts dynamic analysis. The app under analysis is installed on the emulator, and the Unique Identifier (UID) of the analyzed APK is obtained. DLCDroid parses log messages from logcat, presented in JSON format, which contain parameters such as stack, operation, UID, method and classes (optional), and output and source (optional). Messages generated by the app are chosen based on the UID field, and DLCDroid records outcomes and completes its operation if the user ends the analysis.

DLCDroid’s technique for analyzing reflection initiation events, such as the newInstance() method, involves extracting class names and method signatures invoked through reflection. The stack-trace is obtained from the message, and the initial occurrence of a reflection invocation within the stack is identified. DLCDroid then compares this method with the set of Methods of Interaction (MOIs) from the app’s runtime. Once the method is found, DLCDroid integrates the data into the CFG and removes it from the set of undiscovered invoking MOIs.

DLCDroid retrieves the file’s originating address containing the dynamically loaded code (DLC) based on data from the app, downloads the file, and processes it. The file’s hash is calculated, and it is transferred to the results directory with a modified filename incorporating the hash value. This prevents the reexamination of previously investigated code. DLCDroid conducts source code analysis for MOIs within the DLC, and this method is responsible for identifying and categorizing dynamically loaded class invocation details.

DLCDroid can execute on either a physical device or a software-based emulator, offering convenience but potentially encountering emulator-related limitations. To observe dynamic behavior facilitated by DLC, DLCDroid intercepts various Android API functions serving as interfaces to reflection and DLC functionalities. Native method hooking functionalities are incorporated into DLCDroid using inline hooking. The emulator used by DLCDroid is platform-agnostic and capable of intercepting custom code across native operations and Java functions within the Android system, ensuring compatibility with different Android versions. DLCDroid communicates data about DCL and reflection events, such as method calls and class construction, through messages in JSON format, capturing relevant stack trace information and MOI details. In the following subsections, all the phases are detailed in more detail.

### Determining the DLC code

We identify whether the app’s code contains reflection APIs since reflection-induced leaks are the primary focus of our research. To detect the presence of reflection APIs, we employ AndroGuard^32^, a Python-based static analysis framework that works with .apk files. We only proceed with the analysis of applications that AndroGuard has identified as containing reflection APIs.

### Modify emulator and execute apps

In this step, the apps to be analyzed are executed in the modified emulator to make them resistant to environment-aware malware techniques. While the app is running, the logcat file captures the specified tags and logs. The portion of the logs that matches the examples provided in Listings 4.3 to 4.6 shows that the tags REF_CALL and WRAP_API are used to log the methods and class names of reflection APIs. DLCDroid achieves the resolution of reflection calls with run-time dependencies by monitoring during execution analysis.

Dynamic analysis is employed to access the dynamically loaded code during execution and to augment the app’s Control Flow Graph (CFG). In the proposed approach, dynamic assessment occurs on a modified emulator that intercepts API calls through a vtable tampering scheme. DLCDroid logs every event whenever the app initiates a call using reflection or dynamically loads code. These events are accompanied by additional data, such as details regarding the methods being called and their arguments, including the stack-trace in the context of a reflection call. In the context of a DLC call, both the location of the DLC code and the stack trace are provided, and all this data is gathered through the Android log file.

### Tag, configure and log reflection API

In this stage, the app is configured to log its usage of reflection APIs in the Android logcat while it is running. These APIs are logged along with information about the method and class names involved. To facilitate the analysis of repackaged apps, we employ APIMonitor^33^. This code inserts instructions into the app to report the usage of the APIs listed in its configuration file. In order to monitor these reflection APIs effectively, we utilize APIMonitor and modify the reflection APIs in its configuration file.

When the repackaged apps are running, APIMonitor logs the reflected APIs and their arguments and returns results that include a specified tag. We set the tag REF_CALL in the scripts file to log all predefined reflection APIs. It is necessary for us to report both the wrapper API (class and method) and the reflection API. However, one limitation of APIMonitor is that it does not provide information about the wrapper (method and class). To address this limitation, we initially instrument the app using the sootmodule^34^. This allows us to record the reflection API along with the associated wrapper (method and class) using the specified tag WRAP_API.

### Parsing and analysing log file

The log parsing process examines each line of the log and searches for the two specified tags, WRAP_API and REF_CALL. When it encounters the WRAP_API tag, it saves the matching wrapper method and class to be used as the primary key in a hash map and retains the line number in an internal variable. Subsequently, if the log entry with the REF_CALL tag is found, all necessary data is extracted.

DLCDroid analyzes the data collected from the log files. When a reflection call is made, an additional edge is added to the app’s Control Flow Graph (CFG), linking the node of the method that invoked the process through reflection. When DLC is activated, DLCDroid records the file’s location, downloads the file, which includes the DLC code, and performs static analysis. The gathered data is then integrated into the app’s CFG. Furthermore, DLCDroid examines every downloaded file to determine if it contains additional Methods of Interaction (MOIs). The stack-trace information for both the reflection and DLC scenarios is used to determine which MOI initiated the call.

### Instrumentation of DLC code and generate CFG

DLCDroid’s crucial aspect is the generation of instrumented apps by replacing the invocation of reflection methods with identical non-reflection APIs. Modern static analyzers with taint analysis capabilities should be capable of detecting breaches in these instrumented applications. Since the app’s source code is unavailable, instrumentation is carried out in an intermediate representation of the Jimple code within the Soot framework. Initially created for Java bytecode analysis, Soot has been adapted for Android.

The IR code is well-suited for instrumentation due to its representation in a three-address code form with just fifteen different operations. Following instrumentation, the original reflection calls are retained to ensure that subsequent static analysis approaches are unaffected. The key challenge lies in creating non-reflection calls while maintaining data flows. This implies that the outcomes of a non-reflection API should be assigned to an identical variable as the outcomes of a corresponding reflection statement. Additionally, as described in the previous section, the arguments of a non-reflection expression must match the arguments of the equivalent reflection call. Therefore, instrumentation employs both forward and reverse data-flow analysis methods on Jimple code to unpack each parameter within an object’s array.

Generating the Control Flow Graphs (CFGs) for the apps is the next step. CFGs illustrate the connections between invoked methods, and DLCDroid is then used to analyze the apps, including the reflection code. We search for the APIs that the app calls through reflection across each resulting CFG. We experiment with two types of tools: Androguard and SAAF (Soot Android Analysis Framework). While the Androguard approach analyzed all the apps, it requires proper recognition of the reflection API. Results from SAAF often outperform those from Androguard. SAAF can accurately identify the targets of reflection calls. However, when arguments are composed of numerous strings within the apps, encrypted strings, or extracted from a hashtable, SAAF may fail to identify the targets in those situations. DLCDroid proves to be more effective than SAAF because it introduces a dynamic aspect to address the reflection API. Results from the other apps, as indicated by “DLCDroid” reveal that DLCDroid correctly identifies each of the methods invoked using reflection.

### Reveal concealed malicious behaviour

Permissions protect many Android APIs, and these permissions must be explicitly declared in the AndroidManifest.xml file to mitigate potential harm to the Android operating system or user data. We classify an app’s behavior patterns as potentially harmful based on these permissions. Some applications dynamically load code containing API calls protected by permissions. Malicious apps can exploit this strategy to evade detection by static analyzers because the security-sensitive API is dynamically loaded. These apps use reflection APIs to invoke methods secured with risky permissions. The names of methods used to deliver malicious messages are encrypted and decoded only during execution, making it impossible for static analyzers to detect malicious SMS sent using this technique. DLCDroid identifies and raises warnings if these patterns are detected during its investigation. Additionally, we conduct further analysis on the arguments provided to methods invoked through reflection APIs. Suspicious patterns, such as reflective calls to Android APIs protected by unsafe permissions, along with suspicious inputs like the sendTextMessage() method with premium number parameters, assist in identifying concealed malicious behavior via reflection APIs.

## Result analysis

In this Section, we present an overview of our application’s sample suite and share the results from our experimental analysis. DLCDroid underwent evaluation using a dataset comprising authentic benign and malicious applications derived from real-world scenarios. The server and emulator ran on a computing device equipped with 16-core Intel Core i7-13700HX Processor operated at 5.0 GHz clock speed, 30 MB Intel Smart Cache supported by 16 GB LPDDR5X RAM and 1 TB PCIe 4.0 SSD memory.

The assessment dataset included a total of 38,344 applications, with 25,036 classified as benign and 13,308 classified as malicious, belonging to 211 distinct malware families. The benign applications were selected based on their prevalence on the Google Play marketplace. To compile our comprehensive dataset, we sourced malware samples from various reputable repositories, including VirusShare^35^, VirusTotal^36^, Contagio Minidump^37^and use IEEE DataPort service. In addition to downloaded applications, our research involved experimenting with a variety of malicious app samples spanning from 2010 to 2023. These samples were sourced from well-established datasets such as Genome^38^, DREBIN^39^, AndroTracker^40^, SAPIMMDS^41^, AndroProfiler^42^, AndroZoo^31^, CICAAGM2017^43^, AMD^44^, MalDozer^45^, CICAndMal2017^46^, AOM^47^, CDFG^48^and CICMalDroid2020^49^.

Fig. 4 illustrates the frequency of dynamic code application programming interface (API) updates within the analyzed dataset and DLCDroid’s effectiveness in augmenting control flow graphs (CFGs). The data presented shows the proportion of applications within the combined dataset of malicious and benign applications that utilize invoke, DLC, and newInstance() methods. It is observed that approximately 90% of the applications employ newInstance() and invoke() methods, while about 48% of the applications utilize DLC. This demonstrates a substantial increase in the usage of DLC compared to previous state-of-the-art findings. The growth in DLC utilization can be attributed to the increasing complexity of Android applications.

**Table 1:** Performance evaluation for the detection of top ten malware families by DLCDroid Terminology: DR: Detection Rate, #: No.of Samples.

**Malware Family**	Dangerous Permissions	# CFG Expansion	# MOI	DLCDroid Performance
**AnserverBot**	INTERNET READ_PHONE_STATE	# Nodes 1615 # Edges 2094	# Invoke() 5 # NewInstance() 5 # DLC 6	# Samples 117 # Detected 110 DR 94.1 %
**Base Bridge**	READ_PHONE_STATE INTERNET	# Nodes 1780 # Edges 2333	# Invoke() 5 # NewInstance() 3 # DLC 3	# Samples 113 # Detected 109 DR 96.5 %
**DroidDream**	RECEIVE_BOOT_COMPLETED WRITE_SETTINGS CHANGE_WIFI_STATE INTERNET READ_PHONE_STATE WRITE_EXTERNAL_STORAGE CHANGE_NETWORK_STATE CHANGE_WIFI_STATE INSTALL_PACKAGES	# Nodes 15724 # Edges 20369	# Invoke() 18 # NewInstance() 12 # DLC 5	# Samples 125 # Detected 123 DR 98.4 %
**DroidKungFu**	SET_TIME_ZONE WRITE_SETTINGS BLUETOOTH BLUETOOTH_ADMIN INTERNET READ_PHONE_STATE WRITE_SYNC_SETTINGS MOUNT_UNMOUNT_FILESYSTEMS CHANGE_NETWORK_STATE CHANGE_WIFI_STATE ACCESS_COARSE_LOCATION	# Nodes 21170 # Edges 23590	# Invoke() 14 # NewInstance() 7 # DLC 2	# Samples 103 # Detected 101 DR 98.1 %
**FakeInstaller**	SEND_SMS READ_PHONE_STATE INTERNET READ_CONTACTS ACCESS_FINE_LOCATION RECORD_AUDIO	# Nodes 1220 # Edges 2209	# Invoke() 20 # NewInstance() 12 # DLC 5	# Samples 110 # Detected 104 DR 94.5 %
**FakeNotify**	SEND_SMS	# Nodes 172 # Edges 193	# Invoke() 69 # NewInstance() 10 # DLC 0	# Samples 102 # Detected 99 DR 97.1 %
**GingerMaster**	READ_SMS READ_PHONE_STATE READ_CONTACTS WRITE_EXTERNAL_STORAGE INTERNET	# Nodes 195 # Edges 578	# Invoke() 21 # NewInstance() 8 # DLC 4	# Samples 115 # Detected 110 DR 95.7 %
**Obad**	BIND_DEVICE_ADMIN READ_SMS READ_PHONE_STATE READ_CONTACTS BLUETOOTH_ADMIN VIBRATE	# Nodes 467 # Edges 854	# Invoke() 21 # NewInstance() 12 # DLC 3	# Samples 109 # Detected 106 DR 97.2 %
**Plankton**	INSTALL_SHORTCUT READ_PHONE_STATE INTERNET READ_CONTACTS ACCESS_NETWORK_STATE ACCESS_WIFI_STATE	# Nodes 12075 # Edges 17093	# Invoke() 23 # NewInstance() 16 # DLC 7	# Samples 117 # Detected 109 DR 93.2 %
**SMSreg**	SEND_SMS READ_PHONE_STATE	# Nodes 538 # Edges 952	# Invoke() 193 # NewInstance() 1 # DLC 1	# Samples 118 # Detected 108 DR 91.53 %

Table 1 demonstrate the performance evaluation for detecting commonly found top ten malware families by the proposed framework DLCDroid. The DLCDroid average detection rate of malware families is 95.63%, which is better than state-of-the-art approaches. DLCDroid demonstrated the ability to expand the CFG by at least one node in 80% of the examined applications. This expansion was performed solely through reflection, encompassing malicious and benign apps. The minor percentage increase may be attributed to applications that exclusively utilize reflection as a dynamic code change capability. The impact of DLC on CFG expansion and its implications for dynamic behaviour was investigated using findings from applications that employ DLC.

DLCDroid’s effectiveness is particularly relevant when mobile applications utilize DCL. The data indicates a significantly higher prevalence of potentially harmful permissions. Furthermore, malicious applications experience a substantial expansion of their code base by employing dynamic class loading (DCL). This observation suggests that malicious applications exploit sensitive application programming interfaces (APIs) within the executed code. An examination was conducted on the newly added nodes to verify their compliance with system-level permissions and signature-level authorization.

The analytical findings were further explored to identify potential indicators of malicious behavior exhibited by malware. In practical applications, malicious payloads may be embedded within legitimate applications, with the manifest file altered to conceal the additional permissions required for running the payload. In such cases, the final CFG of the application includes nodes protected by additional permissions not present in the original CFG.

This discussion focuses exclusively on applications that utilize Dynamic Class Loading (DCL). The findings reveal that around 67% of applications did not experience any growth in the number of permissions. Conversely, approximately 33% of applications demonstrated the use of additional permissions in dynamically updated code through DLCDroid.

Furthermore, when conducting a more comprehensive examination of DLC code in potentially harmful apps, a noticeable trend emerges regarding the inclusion of dangerous permissions, such as INTERNET and READ_PHONE_STATE. These permissions often indicate potential destructive capabilities, especially the unauthorized disclosure of private information. It is crucial to highlight that this observed behavior results from activating only a small portion of the overall Methods of Interaction (MOIs). The study’s findings provide empirical support for the notion that malware variants tend to possess a higher number of permission types necessary for dynamically loaded code. Consequently, it is justifiable to classify applications as suspicious when they exhibit an excessive level of privilege.

## Related work

Currently, many highly effective static analysis framework HARVESTER^50^struggle to adequately address the concept of reflection. Bodden et al^51^. propose Java reflection code analysis framework named TAMIFLEX for Java applications, but these methods are not directly applicable to Android applications due to their reliance on runtime instrumentation, a technique not supported by the Android platform.

TaintDroid^52^was one of the initial dynamic assessment frameworks for Android applications, monitoring the flow of information across the execution of a single app. It identifies the leakage of user personal data through network interfaces. DroidScope^53^adheres to this methodology. DroidScope facilitates the emulation of application execution and the tracing of context across various tiers of the Android OS at the bytecode level, native code level, and system API level. During the execution of an application in DroidScope, a security^54–56^ analyst can monitor events at various levels and instrument parameters of invoked methods to identify malicious activity.

Dynamic analysis techniques are particularly challenging to automate because they require replicating extensive interactions between applications, the system, and user interface interactions. Various methodologies have been developed to automate the initiation of UI events, ranging from random event generation to more sophisticated techniques such as AppsPlayground^57^and SmartDroid^58^. Nonetheless, each continues to exhibit numerous constraints regarding the types of events they can manage and their scope of coverage.

Ripple^59^ is an investigative approach that incorporates reflection awareness, taint analysis, and type inference to achieve more robust and reliable results. Ripple has found that static taint analysis alone is insufficient for detecting data leaks in scenarios involving inter-component communication (ICC), unsound library summaries, unmodeled Android services, built-in containers, callbacks, and code obfuscation. However, type inference often generates a significant number of inaccurate warnings, particularly when statically inferring parameter names, such as in cases where the parameter name “IMEI” cannot be resolved statically.

The detection of reflective calls in DroidRA^11^ is modeled as an integrated constant propagation issue using COAL [154]. However, its reliance on continuous string inference proves inadequate when reflection API arguments contain runtime dependencies. For example, when attempting to pass getDeviceId(), DroidRA fails to identify leakages. DelDroid^60^ implements the concept of the principle of least privilege through static analysis, aiming to tackle the problem of excessive privileges in dynamically loaded code. It is worth noting that this issue, addressed by DelDroid, is distinct from the research problem we are currently investigating.

IntelliDroid^61^ possesses the capability to ascertain the exact sequence for injecting inputs and carries out this injection at the device-framework interface, ensuring the preservation of system integrity. $DL^2$^62^effectively recognizes the behaviour, extracts path constraints, and executes malicious code using the Reflection API. SEALANT^63^ conducts compositional security analysis at an elevated level of abstraction.

In recent years, significant research has focused on hybrid analysis techniques to address reflection APIs in the Android operating system. Research such as StaDyna^64^ primarily concentrates on examining and resolving dynamic loading of code (DLC) and reflection through a hybrid technique. The initial step of the static assessment involves constructing the Method Call Graph (MCG) for the program, which is then augmented with supplementary data obtained during runtime. However, this evaluation primarily focuses on quantifying the increased quantity of edges and nodes, without considering potential privacy breach risks. The approach requires a customized version of the Android operating system, making installation and use more complex. Additionally, it is limited to Android OS version 4.1.2 r2, limiting its scalability. The system also lacks a direct means of enhancing the capabilities of established static analyzers, particularly in enabling them to conduct analyses sensitive to reflection.

DLCDroid exhibits several distinctions compared to Stadyna. DLCDroid’s emulator utilizes API hooking by manipulating a vTable as its underlying mechanism, avoiding the need to modify the Android OS, which makes it adaptable to various Android versions. DLCDroid is a software tool that analyzes the arguments supplied to procedures invoked via reflection APIs. In contrast to Stadyna, which requires user engagement for app evaluation, DLCDroid employs a triggering mechanism to fully automate the analysis process. Furthermore, DLCDroid is assessed on a much larger sample of apps, including 38.3K, with 25K being benign and 13.3K being malicious, while Stadyna’s evaluation is based on a smaller dataset of 10 apps, with five benign and five malicious. Table 2 presents a summary of the comparative outcomes involving highly pertinent tools, focusing on their analytical capabilities and accessibility. The data reveals that DLCDroid surpasses its counterparts in support of a wide array of DLC analysis features.

**Table 2:** Comparison with the State-of-the-art Approaches.

**Existing Approaches**	**Type of Analysis**	**Detection Rate (%)**	**Evade Anti-Emulation**	**Analyze ICC**	**Analyze collusion attack**	**Analyze DLC/Reflection**
HARVESTER^50^	Hybrid	90%	$\times$	$\times$	$\times$	$\surd$
SEALANT^63^	Static	93%	$\times$	$\surd$	$\surd$	$\times$
Ripple^59^	Static	85%	$\times$	$\times$	$\times$	$\surd$
IntelliDroid^61^	Hybrid	90%	$\surd$	$\times$	$\times$	$\surd$
DroidRA^11^	Static	87%	$\times$	$\times$	$\times$	$\times$
StaDyna^64^	Hybrid	90%	$\times$	$\times$	$\times$	$\surd$
TAMIFLEX^51^	Static	80%	$\times$	$\times$	$\times$	$\surd$
DelDroid^60^	Hybrid	94%	$\times$	$\surd$	$\surd$	$\surd$
**DLCDroid**	Hybrid	95.6%	$\surd$	$\surd$	$\surd$	$\surd$

## Conclusions

In this paper, we introduced DLCDroid, a methodology combining dynamic and static analysis to assess Android applications in reflection and DLC comprehensively. This approach enables the expansion of an application’s Control Flow Graph (CFG) by capturing additional modules loaded during execution and identifying new processing paths concealed through reflection calls. Our proposed method employs code interposition through the hooking of reflection class methods without requiring modifications to the Android framework or the app itself. The evaluation results revealed that malware applications often exhibit a suspicious increase in dangerous permissions following the introduction of new DLC code. This finding underscores the effectiveness of DLCDroid in identifying and preserving dynamic functionalities used by applications at runtime. The comprehensive analysis conducted in this study demonstrates DLCDroid’s resilience against various runtime challenges, including obfuscation, encryption, and reliance on dynamically loaded code. Additionally, the approach can detect malware that utilizes the Inter-Component Communication (ICC) technique to propagate data breaches across different components within a unified application. The findings highlight the limitations of existing static analyzers in the context of reflection, leading to many false negatives. DLCDroid enhances precision by detecting leaks that may be overlooked by static and dynamic analyzers when used in isolation. Meanwhile, DLCDroid improves the effectiveness of current static analyzers by addressing false negatives associated with the reflection code. Furthermore, it is also essential to acknowledge some future improvements in our approach. Sometimes, test automation modules face difficulty in path traversal due to the numerous paths present in Android applications, potentially leading to the oversight of instances of reflection embedded within these paths. Different iterations can sometimes produce distinct real-time temporal values for specific parameters within reflection APIs. However, it isn’t easy to quantify every possible value of these parameters.

## Data Availability

The datasets analyzed are used during the implementation of the research work method, which is available in the repository. https:github.com/Ratibhan/DLCDroid
